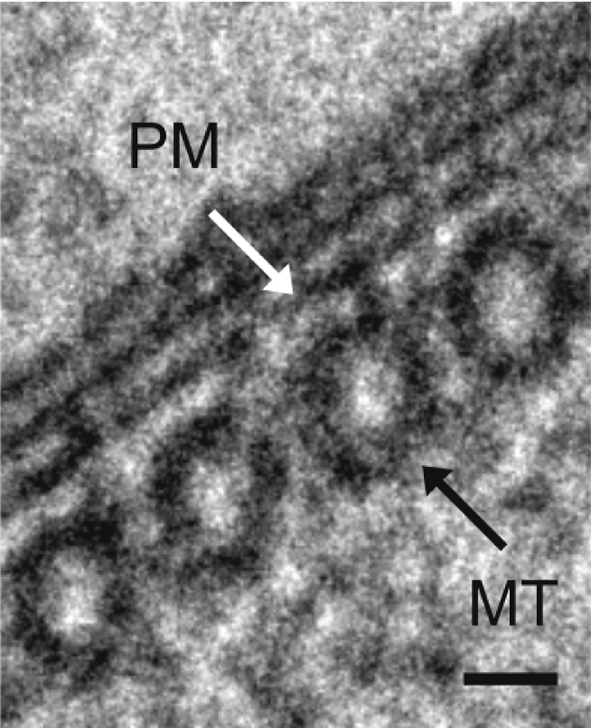# Facilitating trypanosome imaging

**DOI:** 10.1016/j.exppara.2017.03.010

**Published:** 2017-09

**Authors:** Marius Glogger, Ines Subota, Anna Pezzarossa, Anna-Lena Denecke, Mark Carrington, Susanne F. Fenz, Markus Engstler

**Affiliations:** a Department of Cell and Developmental Biology, Biocenter, University of Würzburg, Würzburg, Germany; b Instituto de Medicina Molecular, Faculdade de Medicina, Universidade de Lisboa, Avenida Professor Egas Moniz, 1649-028 Lisboa, Portugal; c Department of Biochemistry, University of Cambridge, Tennis Court Road, Cambridge CB21QW, UK

**Keywords:** Single-molecule fluorescence microscopy, Membrane, Trypanosomes, Hydrogel

## Abstract

Research on trypanosomes as a model organism has provided a substantial contribution to a detailed understanding of basic cellular processes within the last few years. At the same time, major advances in super-resolution microscopy have been achieved, facilitating the resolution of biological structures in living cells at a scale of a few nm. However, the motility of trypanosomes has prevented access to high resolution microscopy of live cells. Here, we present a hydrogel based on poly(ethylene glycol) functionalized with either norbornene or thiol moieties for UV induced thiol-ene crosslinking for the embedding and imaging of live trypanosomes. The resulting gel exhibits low autofluorescence properties, immobilizes the cells efficiently on the nanometer scale and is compatible with cell viability for up to one hour at 24 °C. We applied super-resolution imaging to the inner plasma membrane leaflet using lipid-anchored eYFP as a probe. We find specific domains within the membrane where the fluorescence either accumulates or appears diluted rather than being homogenously distributed. Based on a Ripley's analysis, the size of the domains was determined to be ${\text{r}}_{accumulated}\text{=}\left(170\;\pm \;5\right)$ nm and ${\text{r}}_{dilute}>\left(115\;\pm \;15\right)$ nm. We hypothesize that this structuring of the membrane is associated with the underlying cytoskeleton.

## Highlights

• Super-resolution imaging of motile cells becomes feasible using hydrogel embedding.
• The cytosolic leaflet of the plasma membrane of *T. brucei* is structured.
• Lipid-anchored eYFP preferentially localizes to domains of r = (170 ± 5) nm.
• Lipid-anchored eYFP appears diluted in domains of r > (115 ± 15) nm.

## Graphical abstract

**Figure:**
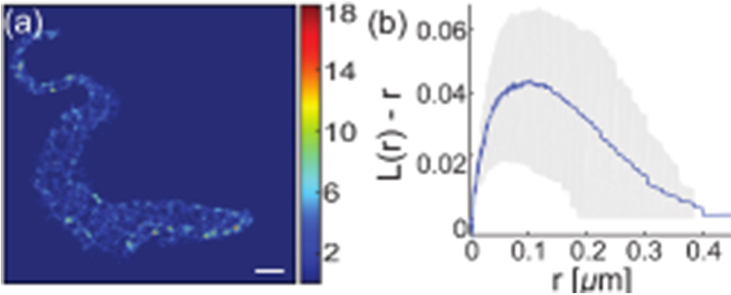

## 1 Introduction

The plasma membrane of trypanosomes can be divided in three regions: the membrane of the cell body, the flagellar membrane, and the membrane of the flagellar pocket. Although the membrane is continuous, the regions serve different functions. While the lipid composition of trypanosomes as a whole is known to be comparable to other eukaryotic cells (Ramakrishnan et al., 2013), the plasma membrane composition has not been studied in isolation. Discussions about the prevalence of membrane structuring elements, such as lipid domains and the potential interaction of proteins, especially the major cell surface component VSG (variant surface glycoprotein), with any domains, are based on *in vitro* experiments and have not reached any certain conclusions (Melkonian et al., 1999, BentingJ et al., 1999, Nolan et al., 2000, Serricchio et al., 2015).

So far the only studies addressing membrane organization *in vivo* (Bülow et al., 1988, Hartel et al., 2015, Hartel et al., 2016) measured the dynamics of either fluorescent lipid tracer molecules or VSG in the plasma membrane of trypanosomes immobilized in agarose or gelatin gels using fluorescence recovery after photobleaching (FRAP). On the time and length scales accessible to FRAP no indications of membrane structuring were found.

Here, we aimed to investigate the existence of membrane organization on the nanometer scale via single-molecule fluorescence microscopy *in vivo*. Only recently, super-resolution microscopy techniques became accessible for highly motile, living trypanosomes using efficient immobilization in cross-linked hydrogels. These novel gels combine gel stiffness and compatibility with cell viability (Glogger et al., 2017).

The dense coat of VSG covering the surface of trypanosomes restricts the access of fluorescent tracers to the plasma membrane. In the past, the coat has been removed by protease digestion to allow access for the tracer (Bülow et al., 1988). However, this approach might interfere with cell integrity and precludes follow-up studies, such as the correlation between VSG and membrane dynamics. Here, we exploited the targeting sequence of the Hydrophilic Acylated Surface Protein B (HASPB), a cell surface protein from *Leishmania major* that is expressed only in infective parasite stages (Flinn et al., 1994). HASPB has an N-terminal sequence of 18 amino acids in the SH4 domain that is necessary and sufficient to target it to the plasma membrane (Denny et al., 2000). This sequence becomes co-translationally myristoylated and post-translationally palmitoylated at the N-terminus. The dual acylation anchors the protein to the inner leaflet of the plasma membrane. HASPB is subsequently translocated across the plasma membrane by an unknown mechanism. When the N-terminal 18 residues from HASPB were fused to GFP (HASPB18::GFP), only 20–30 % was detected on the external face of the plasma membrane in *Leishmania* (Denny et al., 2000). Dual acylation has also been found in the *Trypanosoma brucei* calflagin homologue FCaBP in *Trypanosoma cruzi* (Maric et al., 2011), suggesting a general mechanism in trypanosomes.

In the approach here, the N-terminal 18 amino acid targeting sequence of HASPB fused to eYFP (HASP::eYFP) was used as a marker for membrane structure in the inner plasma membrane leaflet in living bloodstream stage *Trypanosoma brucei*. Single-molecule fluorescence microscopy of the lateral diffusion of HASP::eYFP in the inner leaflet of the plasma membrane allowed us to probe the full membrane area for putative nanometric domains following Ripley's K analysis (Ripley, 1979).

## 2 Material and methods

### 2.1 Cell line

For studies on membrane organization in the cytosolic plasma membrane leaflet *Trypanosoma brucei brucei* Lister 427 13-90 bloodstream form cells were genetically modified to express an eYFP N-terminally tagged with the first 18-amino acid dual acylation sequence from *Leishmania* HASPB protein (Accession number LMJF_23_1060, amino acid sequence: MGSSCTKDSAKEPQKSAD::eYFP; HASP::eYFP, myristoylation and palmytoylation site underlined, respectively). The HASP::eYFP transgene was cloned into pLEW100v5 (Wirtz et al., 1999) so that expression was tetracycline inducible. Single-molecule analysis of membrane organization of the inner leaflet was performed in cells without tetracycline and very low levels of expression to ensure detection of single HASP::eYFP molecules.

For studies on cell immobilization efficiency in cross-linked hydrogels *T. brucei Lister* 427 13-90 containing a constitutively expressed transgene encoding a C-terminally eYFP-tagged kinesin-MORN (Tb927.10.14570) (kinesin-MORN::eYFP) (Glogger et al., 2017) was used.

### 2.2 Cell culture

*T. brucei* bloodstream form cells expressing HASP::eYFP or kinesin-MORN::eYFP were cultivated in HMI-9 medium (Hirumi and Hirumi, 1991) at 37 °C and 5% CO_2_. The medium was supplemented with 10% fetal calf serum containing 5 *μ*g ml^−1^ hygromycin, 2.5 *μ*g ml^−1^ G418 and 2.5 *μ*g ml^−1^ phleomycin (HASP::eYFP) or 0.1 *μ*g ml^−1^ puromycin (kinesin-MORN::eYFP), respectively. The cell density was kept below 5 × 10^5^ cells ml^−1^ to keep cells in the logarithmic growth phase.

### 2.3 Immunofluorescence

Bloodstream stage trypanosomes, expressing HASP::eYFP under control of a tetraycline-inducible promoter, were chemically fixed with 4% paraformaldehyde in 0.1 M HEPES, pH 7.2, for 4 h at 4 °C, washed and resuspended in PBS, 1 %BSA. Rabbit anti-GFP polyclonal antibody (Molecular Probes) was added 1:1000. After 1 h at room temperature, the cells were washed twice with PBS, 1% BSA, and incubated with Alexa594-conjugated anti-rabbit antibody (1:5000) for 1 h. Imaging was done after washing in PBS and addition of DAPI to visualize DNA. An aliquot of the same batch of fixed trypanosomes were permeabilized with PBS, 0.2% TRITON-X100, for 2 min at room temperature.

### 2.4 Preparation of glass cover slips

Glass cover slips (Hecht Assistent, 22 × 22 mm, thickness corrected) were cleaned in 2% Hellmanex II solution in a sonication bath for 15 min at 60 °C followed by copious rinsing with deionized water. A second sonication and rinsing procedure was performed in deionized water and glass cover slips were stored under fresh deionized water for a maximum of three days.

### 2.5 Hydrogel formation

8-arm poly(ethylene glycol, PEG) norbornene (20 kDa, Sigma-Aldrich, Germany) and linear PEG-dithiol (1 kDa, Sigma-Aldrich, Germany) were dissolved in TDB (5 mM KCl, 80 mM NaCl, 1 mM MgSO_4_, 20 mM NaH_2_PO_4_, 2 mM NaH_2_PO_4_, 20 mM glucose, pH 7.6) with equimolar ratios of norbornene:thiol to a total polymer concentration of 10% (w/v). The solution was gently agitated in the dark at 37 °C for 30 min and 0.025% (w/v) photoinititator Irgacure-2959 (I2959, BASF) were added. The pH-value was adjusted to 7–7.5 using 1 M NaOH and the precursor solution was kept on ice. For cell encapsulation, 5 × 10^6^ cells were centrifuged at 1400x*g* for 10 min at 4 °C and washed three times with 1 mL ice-cold TDB. Trypanosomes were carefully resuspended in 10 *μ*l of the precursor solution. The solution was immediately centrifuged at 1000x*g* for 1 min at 4 °C between two glass cover slips mounted in a sample chamber. The hydrogel was then cross-linked using UV-light exposure (365 nm, UVL hand lamp with filter, A. Hartenstein, Würzburg, Germany) for 2 min at 4 °C with an intensity of 1 mW cm^−2^. The immobilization procedure was performed strictly at low temperatures to avoid endocytosis of reactive precursor solutions.

### 2.6 Cell viability assay

Viability of trypanosomes in cross-linked hydrogels was monitored using propidium iodide (PI) as a live/dead fluorescence marker. 5 *μ*g ml^−1^ PI was added to the cell solution containing hydrogel precursors and photoinitiator and immobilization was performed as described before. An inverted wide-field microscope (Leica DMI6000B) was used to detect PI-fluorescence with excitation and emission filters of 530–560 nm and 572–648 nm, respectively. Cell viability was monitored for 60 min at 24 °C. As a control, trypanosomes were prepared in TDB in the absence of photoinitiator or hydrogel precursors.

### 2.7 Single-molecule imaging

An inverted wide-field microscope (Leica DMI6000B) setup equipped with a high numerical aperture lens (HCX PL APO 100×/1.47 OIL CORR TIRF) and an EMCCD-camera (Andor Technology) was used for single-molecule imaging. The image size was set to 120 × 120 pixels with a pixel size of 160 nm. Measurements were done by illumination for 10 ms with a 515 nm laser beam (Cobolt) at an intensity of 2 kW cm^−2^. For trypanosomes expressing HASP::eYFP or kinesin-MORN::eYFP pre-bleaching was performed if necessary until single molecules could be monitored. 8000–10,000 consecutive images were recorded at 28 Hz. Signals were detected by the camera using appropriate filter combinations (zt405/514/633rpc (dichroic, Chroma) and 550/49 BrightLine HC (emission filter, Semrock)). eYFP signals were singled-out by exploiting the intrinsic blinking properties of eYFP at high illumination intensities (Fölling et al., 2008, Lee et al., 2011).

### 2.8 Image processing and data analysis

Image analysis was done using routines written in MatLab (Mathworks Inc., USA).

#### 2.8.1 Localization

Single-molecules were localized with a precision of *σ* = (23 ± 5) nm as described before (Schmidt et al., 1996, Fenz et al., 2012). In brief, individual eYFP signals were fitted with two-dimensional Gaussian profiles of mean full width at half-maximum of (280 ± 41) nm set by the diffraction limit. These results were filtered with respect to the known eYFP single-molecule footprint (peak intensity and peak width) as well as detection error thresholds. Clustering observed in PALM experiments can result from subsequent reactivation of the same, immobile molecules (Annibale et al., 2011). Here, diffusing eYFP proteins served as the basis to scan the inner membrane leaflet and look for areas of preferred occupancy. Thus, no artificial clustering was expected. However, fluorescent contaminations of the hydrogel are immobile and could distort cluster analysis. We introduced two measures to reduce the influence of such contaminations. First, a manually defined mask was implemented to separate the cell from the background. Second, eYFP signals that reappeared within 50 consecutive frames, i.e. within 1.8 s, and were found within the positional accuracy of the first localization, were discarded to remove false-positive localizations on the cell.

The final list of single-molecule positions $\left(x_{i},\;y_{i}\right)$, together with the localization precision $\sigma_{i}$ for each molecule served as the input for further analysis.

#### 2.8.2 Label density and autofluorescent background

The label density on the flagellar axoneme (kinesin-MORN::eYFP) as well as the extent of the autofluorescent background were determined as described previously (Glogger et al., 2017).

### 2.9 Occupation maps

In order to visualize the membrane occupancy, maps reflecting the number of HASP::eYFP per area were calculated from the single-molecule positions. Maps were initialized at increased resolution. Each pixel of the original image was replaced by nine subpixels in the map. This value was chosen from the cumulative distribution of nearest neighbor distances calculated between all single-molecule positions. At the dimension of a subpixel 97% of all proteins possess at least one neighbor. See SI Fig. 1b for the cumulative distribution of nearest neighbor distances. The occupancy was calculated as the number of localizations per subpixel.

### 2.10 Filling of dilute membrane regions

The occupation maps were scanned for regions of dilute HASP::eYFP localizations. For that purpose, each subpixel with a maximum of one localization was labeled as dilute and used to generate a mask. Single subpixels were removed from this mask to reduce noise. The remaining objects were labeled and all except the largest, which corresponds to the background, were artificially populated with 100 evenly spaced localization per subpixel.

### 2.11 Ripley's analysis

To identify potential nonrandom distributions of proteins on the plasma membrane, we used Ripley's L(r)−r function (Ripley, 1979) sequentially on both the original single HASP::eYFP positions and the artificial positions created in formerly dilute areas, following the procedure described in (Pezzarossa et al., 2015). The L(r)−r function yields 0 in case of a random distribution and is positive in case of clustering. A maximum of L(r)−r provides a good estimate for the domain radius (Ripley, 1979).

For the Ripley analysis, the images were divided in square tiles with a side length of 0.8 μm. Typically 60 tiles were fit onto each cell. Only tiles containing at least 50 localizations were processed further. Ripley's L(r)−r function was calculated up to a radius of r = 0.5 μm for each tile and averaged over all tiles and all cells. The mean 〈L(r)−r〉 together with the 1σ - confidence interval is reported.

### 2.12 Super-resolution image and sizing

The super-resolution images of either the axoneme (kinesin-MORN::eYFP) or the cell membrane (HASP::eYFP) were calculated as the sum of all fitted single-molecule positions. Each localization (*x_i_*, *y_i_* ) was plotted as a 2D Gaussian spot with the respective width given by the mean positional accuracy, $\sqrt{\sigma_{x}^{2}+\sigma_{y}^{2}}$. Each image was reconstructed of at least 3 × 10^3^ (kinesin-MORN::eYFP) or 5 × 10^3^ (HASP::eYFP) positions.

The width of the kinesin-MORN::eYFP signal in the super-resolution image was quantified to assess the immobilization efficiency of the hydrogel. This procedure was described in detail in (Glogger et al., 2017).

## 3 Results and discussion

### 3.1 Cell viability in the hydrogel

Propidium iodide (PI) staining of *T. brucei* bloodstream form cells expressing HASP::eYFP was performed to monitor cell viability in cross-linked hydrogels. PI is a membrane-impermeant fluorescent dye, which is excluded from viable cells. Upon loss of cell membrane integrity, PI is able to bind intracellular DNA and becomes fluorescent at an excitation maximum of 535 nm. In a two-color experiment the HASP::eYFP and the PI signal can be unequivocally separated and thus, dead cells can be identified. Cell viability of > 90% was achieved at room temperature in immobilized trypanosomes over 60 min after hydrogel-crosslinking (Fig. 1a, white bars). Similar survival rates were found for trypanosomes in the absence of polymer precursor and photoinititator (Fig. 1a, gray bars).

**Fig. 1:**
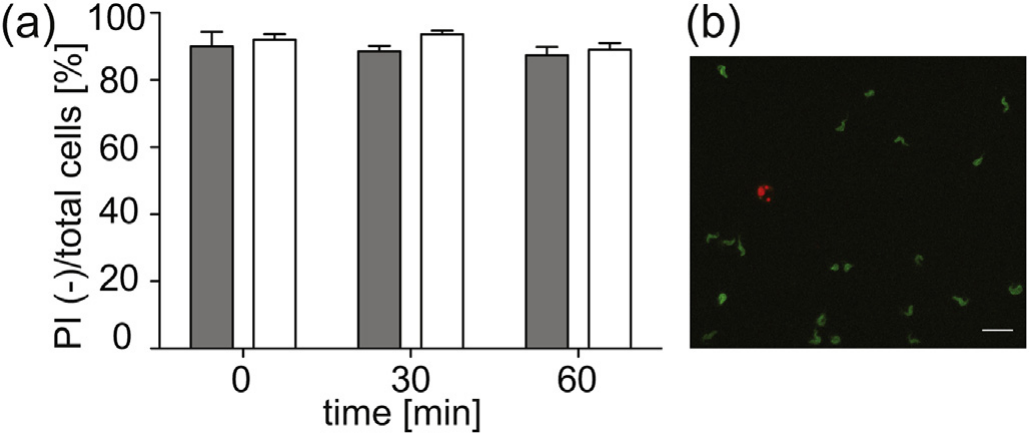
Viability of immobilized trypanosomes in 10% (w/v) PEG-norbornene/PEG-dithiol hydrogels. (a) Survival rate of cells (PI negative (−), mean ± S.D, 3 replicate experiments) over 60 min after cross-linking in hydrogels (white bars) or buffer solution (TDB, gray bars). (b) Overlay fluorescence image of immobilized living (green, HASP::eYFP signal) and dead (red, PI signal) cells. The scale bar is 20 *μ*m.

These results emphasize the mild conditions of thiol-ene cross-linking PEG-norbornene and PEG-dithiol in the cell embedding process and high cytocompatibility after polymerization for 60 min. Monitoring cellular viability over longer time scales was not performed, as a negative effect of complete immobilization on cell function cannot be excluded. This concern is substantiated by the finding that trypanosomes show a reduced viability in the hydrogel 40 min after immobilization at 37 °C (data not shown). Nevertheless, the available time window was sufficient for live cell microscopy. Hydrogel systems based on thiol-ene click chemistry have been used for encapsulation of human mesenchymal stem cells in allyl- and thiol-functionalized linear poly(glycol) (Stichler et al., 2016) or norbornene- and dithiol-functionalized PEG as well as norbornene functionalized hyaluronic acid cross-linked with di-thiols (Gramlich et al., 2013). Thiol-ene based hydrogels are tunable with respect to gel stiffness (Le et al., 2016) and possess minor heterogeneity within their structure (Shih and Lin, 2012), which make them suitable for immobilization of motile cells like trypanosomes (Glogger et al., 2017). Further, photo initiator I2959 has minimal toxicity for many mammalian cell types (Williams et al., 2005) and propidium iodide does not impede cell viability of *T. brucei* cells at low concentrations (Gould et al., 2008). In summary, immobilization of *T. brucei* expressing HASP::eYFP in PEG-norbornene/PEG-dithiol hydrogels allows imaging of viable cells on time scales up to one hour at room temperature.

### 3.2 Immobilization efficiency of trypanosomes in the hydrogel

The immobilization efficiency of PEG-norbornene/PEG-dithiol hydrogels was analyzed by recording super-resolution images of embedded live trypanosomes expressing kinesin-MORN::eYFP. The fusion protein localized to the 9 + 2 axonemal structure inside the flagellum (Glogger et al., 2017). Imaging this structure allowed an analysis of the immobiliziation efficiency on the nanometer scale as residual flagellar beating results in measurable spreading of the signal and thus, in an increased apparent axonemal diameter.

Super-resolution images were constructed from the positions of blinking eYFP molecules. A mean localization precision of *σ* = (23 ± 5) nm could be achieved for single eYFP molecules in embedded trypanosomes. Next to specific signals in the shape of the flagellum, homogeneous background originating from the gel and intracellular fluorescence signals originating from both autofluorescence of the cell and eYFP proteins being translated and transported, were detected (Fig. 2a). Comparison of the signal density on and off the structure of interest, allowed us to quantify the combined contribution of false-positive signals as described before (Glogger et al., 2017). We found that this contribution was generally very low (≈ 1%, see SI Fig. 2a) and could therefore be neglected. At the same time, the high label density on the flagellum ensured good sampling of the structure. It ranged from 450 molecules *μ*m^−2^ to 1300 molecules *μ*m^−2^, with an average of 800 molecules *μ*m^−2^ (see SI Fig. 2b). In summary, the high specific label density and good signal-to-noise ratio facilitates super-resolution microscopy of trypanosomes inside the hydrogel.

**Fig. 2:**
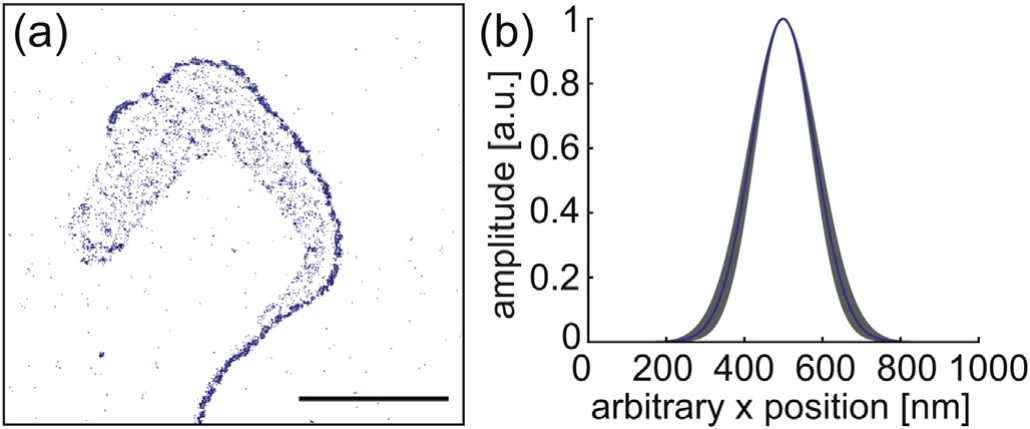
Super-resolution imaging of the axonemal structure in immobilized, living trypanosomes. (a) Super-resolved image of a kinesin-MORN::eYFP expressing cell in a PEG-norbornene/PEG-dithiol hydrogel. The scale bar is 5 *μ*m. (b) Mean cross section of the kinesin-MORN::eYFP at the axoneme of immobilized trypanosomes. The blue line depicts the mean axonemal cross section and the shaded region the S.D.

The diameter of kinesin-MORN::eYFP signals was measured in super-resolution images of 34 immobilized trypanosomes. An axonemal diameter of (191 ± 23) nm was determined by fitting the mean intensity profile perpendicular to the flagellar structure (Fig. 2b). Similar results were found for chemically fixed trypanosomes (Glogger et al., 2017) and in electron micrographs (Koyfman et al., 2011, Nicastro et al., 2006). This good agreement between the measured axonemal dimension in fixed and living trypsanomes as well as in electron micrographs proves that trypansomes are very efficiently immobilized in 10% (w/v) PEG-norbornene/PEG-dithiol hydrogels.

### 3.3 HASP::eYFP distribution in the plasma membrane of trypanosomes

**Fig. 3:**
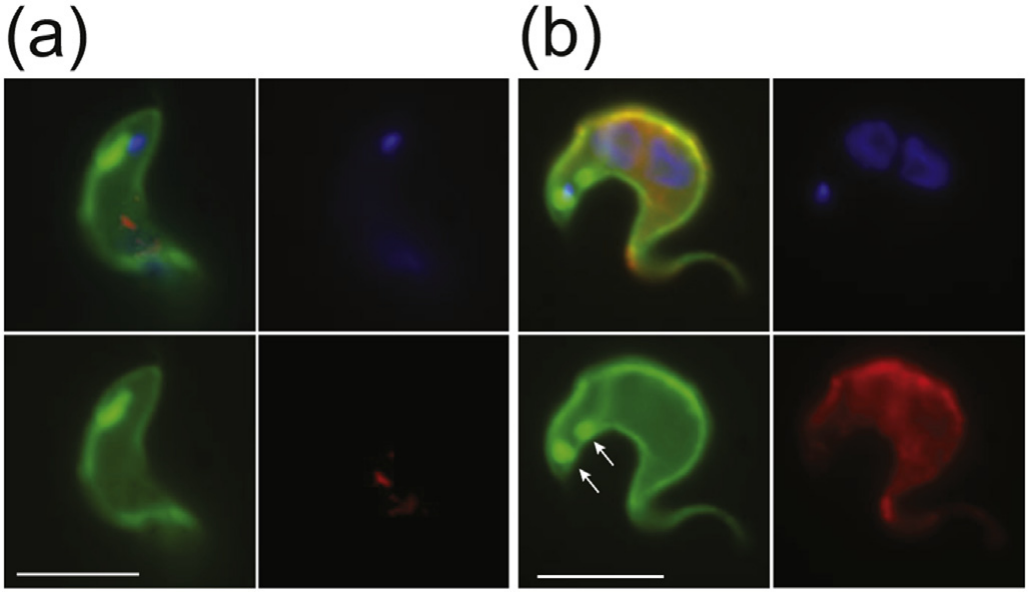
HASP::eYFP is targeted to the inner leaflet of the plasma membrane. Immunofluorescence images of HASP::eYFP expressing cells using anti-GFP polyclonal antibodies in paraformaldeyde-fixed (a) and further permeabilized cells (b). Green, YFP-fluorescence; red, anti-GFP; blue, DAPI. The upper left corner of each panel displays the overlay of all color channels. The HASP-eYFP molecules inside the duplicated flagellar pocket (arrows) are not accessible to the anti-GFP antibodies under the conditions used. The scale bar is 5 *μ*m.

**Fig. 4:**
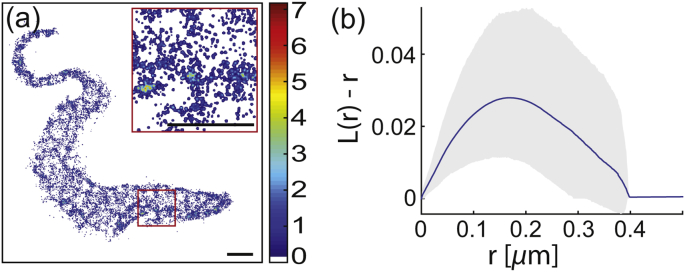
HASP::eYFP distribution in the plasma membrane of *T. brucei*. (a) Reconstructed super-resolved image of HASP::eYFP. An enlarged version of the area inside the red rectangle is shown in the upper right corner. The scale bar is 1 μm. (b) Ripley's L(r)−r function averaged over 17 cells (blue line) together with the 1*σ* confidence interval (gray area). The function has a maximum at $r_{accumulated}=\left(170\;\pm \;5\right)$ nm.

In light of the good retention of viability and excellent immobilization efficiency, the applicability of the PEG-norbornene/PEG-dithiol hydrogel for live cell super-resolution imaging was tested. As a proof of principle, we recorded single HASP::eYFP molecules diffusing in the inner leaflet of the trypanosomal plasma membrane. Targeting of HASP::eYFP to the inner leaflet was assessed by immunofluorescence (Fig. 3). We hypothesize that the mechanism responsible for the translocation to the outer leaflet in *L. major* is absent in *T. brucei*. We visualized the cytosolic membrane leaflet by reconstructing the super-resolution images from all collected HASP::eYFP localizations to detect potential membrane structuring. One example is displayed in Fig. 4a together with a close up view of a small membrane patch. While the fluorescent tracer explores the full plasma membrane including the flagellar pocket and flagellar membrane, apparent preferred domains within these region are visible by eye and analysis of the nearest neighbor distances (NND) supports this observation. See SI Fig. 1 for the histogram of NND probabilities and the cumulative distribution. The most frequent NND was 14 nm, while from the number of single-molecule localizations (266363 on 17 cells) and the known surface area of a trypanosome (≈ 100 *μ*m^2^, but note that only one half was imaged), we would expect a distance of 65 nm, if the HASP::eYFP molecules were evenly distributed on a hexagonal grid. Ripley's analysis was performed to quantify the observed pattern. The L(r)−r function exhibited a clear maximum at a cluster radius of $r_{accumulated}=\left(170\;\pm \;5\right)$ nm (Fig. 4b). The width of the 1*σ* confidence interval and the associated range of cluster radii $r_{accumulated}:\;140–205$ nm is quite broad as expected for domains that deviate from the circular shape inherently assumed in the Ripley's analysis.

**Fig. 5:**
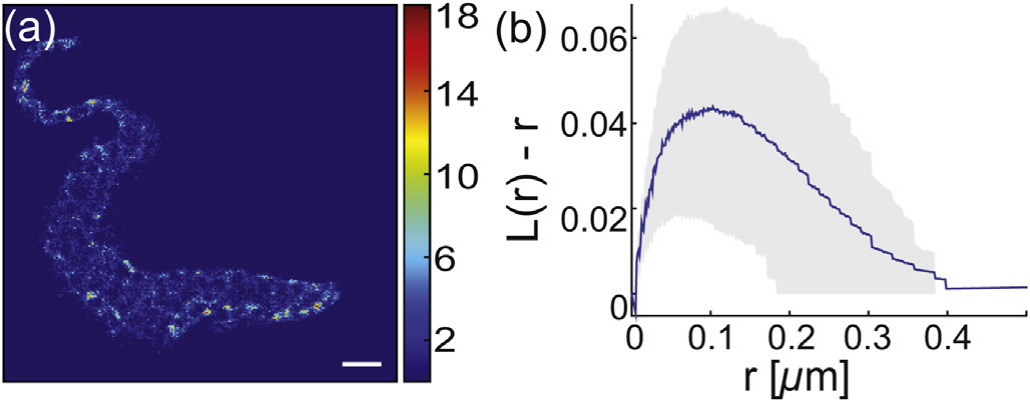
Diluted membrane areas. (a) Occupancy map of HASP::eYFP in the plasma membrane of *T. brucei*. The scale bar is 1 μm. (b) Ripley's L(r)−r function calculated for the artificially filled dilute membrane regions (blue line) together with the 1*σ* confidence interval (gray area). The function has a maximum at r = (115 ± 15) nm.

In addition to the areas of preferred appearance, the coarse-grained occupation map reveals that in the same cell there are dilute areas of similar size (dark blue patches on the cell in Fig. 5a). Thus, the Ripley analysis above reports a net effect of accumulation and dilution with the accumulation being the dominant contribution. In order to separately analyze the devoid areas a detour was implemented. Dilute areas in the occupation maps were identified and artificially populated with localization coordinates to make them accessible to Ripley's analysis. The L(r)−r function calculated from these coordinates exhibited a clear maximum at a domain radius of $r_{dilute}=\left(115\;\pm \;15\right)$ nm (Fig. 5b). As a control of our analysis approach, the non-devoid areas were treated the same way resulting in $r_{control}\;\approx$ 130 nm. This value is smaller than the result based on the raw data ($r_{accumulated}$ = (170 ± 5) nm), but within the range. Thus, we conclude that the approach of artificially filling devoid area underestimates the corresponding domain radius and $r_{dilute}>$ (115 ± 15) nm.

Although the dual acylation of HASP::eYFP results in a marker for ordered lipid domains (Lorent and Levental, 2015, Levental et al., 2015), we do not interpret the observed segregation in light of the lipid raft model. Both the spatial scale of several hundred nanometers and the stability over the time of the measurement (5 min) provide strong arguments against the current view of lipid domains as small, highly dynamic structures (Pike, 2006). Given the methodology of this study we can neither prove nor disprove the existence of lipid domains in trypanosomes.

One possible explanation for the irregular distribution of HASP::eYFP is that it is caused by potential interactions with the cytoskeleton as described by the fences and pickets model (Kusumi et al., 1993) with a few modifications. First, trypanosomes rely on a microtubule corset replacing actin in the model. The distance between the microtubules and the plasma membrane measured in electron micrographs amounts to (14 ± 4) nm (Supplementary Material and Methods and Supplementary Fig. 3) and is thus substantially larger than the diameter of the YFP barrel (< 5 nm (Ormoe et al., 1996)). As a consequence, we do not expect direct interactions between HASP::eYFP and the microtubule corset. Nevertheless, the regularly-spaced subpellicular microtubules of the trypanosome cytoskeleton are highly organized. They are linked to each other and to the plasma membrane by microtubule associated proteins (Kohl and Gull, 1998). Second, from the data here, we cannot distinguish between pickets that are either transmembrane proteins or membrane anchored or both.

To test and refine our hypothesis we plan (i) improved tracking with a stable label to look for hop-diffusion, (ii) two-color experiments to simultaneously observe diffusion in the membrane and cytoskeletal structures, and (iii) diffusion measurements in the outer membrane leaflet to clarify the nature of the pickets as well as to search for signs of interleaflet coupling.

## 4 Conclusion

We present UV-crosslinked PEG-norbornene/PEG-dithiol hydrogels as a readily available embedding method allowing for high-resolution, live-cell imaging of trypanosomes. The hydrogel efficiently immobilizes the cells while maintaining cell viability for up to one hour. Thus, slow imaging techniques like fluorescence-based super-resolution become feasible as shown before (Glogger et al., 2017). The combined autofluorescent background of the gel and the cells is very low allowing for single-molecule imaging. As a proof-of-principle, we present a super-resolution image of the inner plasma membrane leaflet from trypanosomes. We find that HASP::eYFP is not homogeneously distributed across the membrane, but exhibits both accumulation in certain domains and dilution in other domains. We hypothesize that this structuring of the membrane is associated with the underlying cytoskeleton.

## Figures and Tables

**Fig1 SI:**
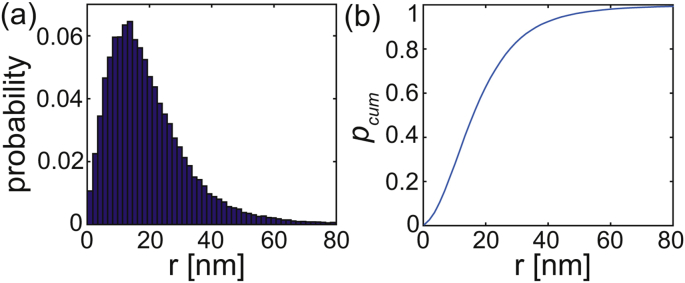


**Fig2 SI:**
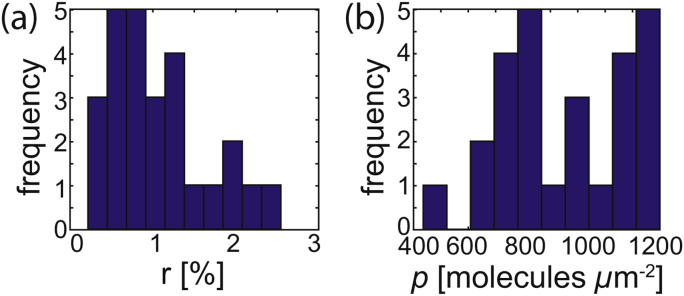


**Fig3 SI:**