# Supplementary material for: Facilitating trypanosome imaging
Source: Exp Parasitol. 2017 Sep;180:13–8. doi: 10.1016/j.exppara.2017.03.010 (PMC5540225; doi:10.1016/j.exppara.2017.03.010)
Supplement: Supplementary material [file mmc1.docx]

1. Supplementary Information

1.1. Supplementary Material and Methods

1.1.1. High Pressure Freezing and Freeze Substitution

The high pressure freezing protocol was developed based on work by Höög et al. [1]. 2 x 10 bloodstream form *T. brucei* in 50 ml HMI-9 medium were concentrated by centrifugation. Cells were centrifuged twice at 750 x g and room temperature for 3 min. After the first centrifugation the volume was reduced to 4 ml and supplemented with heat-inactivated FCS as a cryoprotectant at a final concentration of 50 % (v/v). After the second centrifugation 200 μl of the cell suspension was transferred to a minifuge and further compacted for 5 s. 2 μl of the final cell pellet was transferred to the freezing container. High pressure freezing in a EM HPM100 (Leica Microsystems) was carried out at a freezing speed $>$ 20,000 K s and a pressure $>$ 2100 bar. The samples were stored in liquid nitrogen until freeze substitution in an EM AFS2 freeze substitution system (Leica Microsystems). First, they were incubated in anhydrous acetone containing 0.5 % (v/v) glutaraldehyde and 0.1 % tannic acid (v/v) at 90 C for 24 hours. The solution was renewed and the incubation continued. After in total 96 hours of incubation, the cells were washed four times with anhydrous acetone at -90 C for 4-6 h followed by incubation in 2 % osmium tetroxide (w/v) for 28 h at -90 C. The temperature was ramped to -20 C over 14 h, kept constant for 16 hours and ramped to 4 C over 4 additional hours. After four more washing steps with anhydrous acetone within 2 hours the temperature reached room temperature after one more hour.

1.1.2. Embedding and Sectioning

The cells were removed from the EM AFS2 and incubated in 50 % epon in acetone for 5 h at room temperature followed by incubation in 90 % epon (v/v) in acetone overnight at 4 C. The solution was exchanged for 100 % epon at room temperature every 2-3 hours for 9 hours in total before starting the polymerization at 60 C for 24 hours. The embedded cells were cut into sections of 55 nm (Diatome AG).

1.1.3. Contrasting and Transmission Electron Microscopy

For contrasting, the sections were incubated in 2 % uranyl acetate (v/v) for 15 min and washed three times in decocted ddH2O followed by incubation in 50 % Reynolds lead citrate (v/v) for 5 min. The sections were washed twice in decocted ddH2O and allowed to dry. TEM images were acquired using a transmission electron microscope (JEOL JEM-2100) at an acceleration voltage of 200 kV.

1.2. Supplementary Figures

Figure 1: (a) Histogram of the nearest neighbor distances between HASP::eYFP molecules based on 17 cells. (b) Cumulative distribution of the nearest neighbor distances.

Figure 2: Optical properties of the embedded cells expressing kinesin-MORN::eYFP. (a) Histogram of the label density ρ in the flagellar region.(b) Histogram of the percentage of unspecific signal at the flagellum r = $\rho_{BG}$/ρ.

Figure 3: Electron micrograph featuring trypanosomal microtubules (MT) and the plasma membrane (PM). The distance d = 14 $\pm$ 4 nm between both structures was determined from the midpoint of the respective osmium stains for 50 MTs. The scale bar is 20 nm.

1.3. Supplementary References

[1] Höög JL, Gluenz E, Vaughan S and Gull K 2010 *Methods Cell Biol.* **96** 175-196
